# Orange is the new black: Kinases are the new master regulators of tumor suppression

**DOI:** 10.1002/iub.1981

**Published:** 2018-12-11

**Authors:** Elvira An, John Brognard

**Affiliations:** ^1^ Laboratory of Cell and Developmental Signaling, Center for Cancer Research National Cancer Institute Frederick MD

**Keywords:** tumor suppressors, kinases, signal transduction, LKB1, MKK4, MLK4, DAPK3, PKC, Eph receptors

## Abstract

For many decades, kinases have predominantly been characterized as oncogenes and drivers of tumorigenesis, because activating mutations in kinases occur in cancer with high frequency. The oncogenic functions of kinases relate to their roles as growth factor receptors and as critical mediators of mitogen‐activated pathways. Indeed, some of the most promising cancer therapeutic agents are kinase inhibitors. However, cancer genomics studies, especially screens that utilize high‐throughput identification of loss‐of‐function somatic mutations, are beginning to shed light on a widespread role for kinases as tumor suppressors. The initial characterization of tumor‐suppressing kinases— in particular members of the protein kinase C (PKC) family, MKK4 of the mitogen‐activated protein kinase kinase family, and DAPK3 of the death‐associated protein kinase family— laid the foundation for bioinformatic approaches that enable the identification of other tumor‐suppressing kinases. In this review, we discuss the important role that kinases play as tumor suppressors, using several examples to illustrate the history of their discovery and highlight the modern approaches that presently aid in the identification of tumor‐suppressing kinases. © 2018 IUBMB Life, 71(6):738–748, 2019

**Abbreviations**
AKT: Protein kinase B
AMPK: Adenosine monophosphate activated kinase
ATM: Ataxia‐telangiectasia mutated kinase
CAMPKK2: Ca^2+^ − and calmodulin‐dependent protein kinase kinase 2
CCLE: Cancer Cell Line Encyclopedia
CHEK2: Checkpoint kinase 2
CRC: Colorectal cancer
CREB: cAMP response element‐binding protein
CRTC: CREB‐regulated transcription coactivator
DAG: Diacylglycerol
DAPK: Death‐associated protein kinase
EGFR: Epidermal growth factor receptor
Eph: Erythropoietin‐producing hepatoma amplified sequence
Ephrin: Eph receptor‐interacting protein
ERBB: Erythroblastic oncogene B (Her)
GOF: Gain‐of‐function
GPI: Glycosylphosphatidylinositol
HDACs: Histone deacetylases
JNK: Jun‐N‐terminal kinase
JUN: Transcription factor AP‐1
LKB1 (*STK11*): Liver kinase B1
LOF: Loss of function
LOH: Loss of heterozygosity
MARK: Microtubule‐affinity‐regulating kinase
MKK4 (*MAP2K4*): Mitogen‐activated protein kinase kinase 4
MLC2: Myosin light chain 2
MLK4 (*MAP3K21*): Mixed lineage kinase 4
MSS: Microsatellite stable
mTOR: Mammalian target of rapamycin
MYPT1: Myosin phosphatase targeting‐1 protein
NUAK: NUAK family SNF‐1‐like kinase
PI3K: Phosphatidyl‐inositol 3‐kinase
PJS: Peutz‐Jeghers Syndrome
PKC: Protein kinase C
PP1β: Serine/threonine protein phosphatase
PPAR*γ*2: Peroxisomal proliferator‐activated receptor *γ*2
RAF: Rapid accelerator of fibrosarcoma
RAS: Rat sarcoma GTPase
SIFT: Sorting intolerant from tolerant
SIK: Salt inducible kinase
siRNA: Silencing RNA
T‐PLL: T‐cell prolymphocytic leukemia
TCGA: The Cancer Genome Atlas
TP53: Tumor protein p53
TSC2: Tuberous sclerosis 2 protein
v‐fps: Tyrosine‐protein kinase transforming protein Fps
WT: Wild type

## INTRODUCTION: SHIFTING THE PARADIGM FROM TUMOR PROMOTING TO TUMOR SUPPRESSING

Protein kinases are members of a large family of enzymes that phosphorylate and modulate the activity, binding partners, and localization of their target protein substrates. The dynamic regulation of phosphorylation is a key mechanism to control cell proliferation, migration, and cell survival, which are processes associated with normal physiology, tumorigenesis, and cancer progression. By binding and stimulating the activity of receptor tyrosine kinases, mitogenic factors, such as growth factors, robustly activate both the receptors, which are kinases, and mitogenic signaling cascades, which are also regulated by kinases. The importance of kinases in promoting tumorigenesis is represented by the numerous examples of over‐activation of signal transduction cascades by kinases harboring gain‐of‐function (GOF) mutations. Thus, kinases can be key oncogenic drivers. However, kinases can also be tumor suppressors, either acting solely as a tumor suppressor or acting as both a tumor promoter and tumor suppressor, depending on the genetic make‐up of the tumor. Therefore, the historical paradigm of kinases as oncoproteins is being overturned.

Here, we take a historical perspective on the discovery of tumor‐suppressing kinases and highlight lessons that we have learned from highly studied tumor‐suppressing kinases. We begin by describing the kinases first identified with tumor‐suppressing activity, detail the distinct mechanisms by which kinases suppress tumorigenesis, and end with examples illustrating the modern approaches used to identify tumor‐suppressing kinases.

### LKB1: A Tumor‐Suppressing Kinase Is Born

One of the first kinases characterized as a tumor suppressor was liver kinase B1 (LKB1). LKB1 is a conserved serine–threonine kinase encoded by *STK11* gene, which is located on human chromosome 19p 1. LKB1 forms a heterotrimeric complex with the pseudokinase STE20‐related adaptor (STRAD*α*, encoded by *STRADA*) and the scaffolding protein 25 alpha (MO25*α*, encoded by *CAB39*) 2, 3, 4, which are both required for LKB1 activation and downstream activation of adenosine monophosphate (AMP)‐activated kinase (AMPK) and AMPK‐related kinases 5. Studies with AMPK catalytic subunit isoforms (AMPK*α*1 and AMPK*α*2) show that LKB1 phosphorylates the conserved T‐loop Thr residue (Thr 172), which is required for AMPK activation 5.

The potential tumor‐suppressive activity of LKB1 was identified in 1997: truncation mutations in LKB1 were found to cause Peutz‐Jeghers Syndrome (PJS) 6, which is an inherited intestinal polyposis syndrome 7. Patients with PJS have a high risk of developing cancer. Characteristic of the early discoveries of tumor‐suppressing genes, *STK11* was discovered through studies that pinpointed truncating germline mutations in a gene residing on chromosome 19p in multiple individuals affected by PJS. Specifically, the locus for PJS was mapped through comparative genomic hybridization and genetic linkage analysis 8. Loss of heterozygosity (LOH) at the *STK11* locus in numerous tumor types also supported a tumor‐suppressive function for LKB1 9. Somatic loss‐of‐function (LOF) mutations in *STK11* occur in sporadic cancers 10, and mice with heterozygous LOF mutations of *STK11* develop gastrointestinal hamartomas that mimicked the PJS phenotype.

Patients with PJS predominantly develop hamartomatous polyps that are generally benign, indicating LOF mutations in LKB1 predispose these patients to cancer but that additional mutations in other genes are required for the development of a malignant phenotype. Indeed, PJS patients have a high risk of developing gastrointestinal tumors and lung cancers 11, 12, on accumulation of subsequent driver mutations. Additionally, LKB1 is an important tumor suppressor in adenocarcinomas, specifically non‐small cell lung cancer adenocarcinomas, where LKB1 is mutated in 33% of all cases 13, 14, 15.

**Figure 1:**
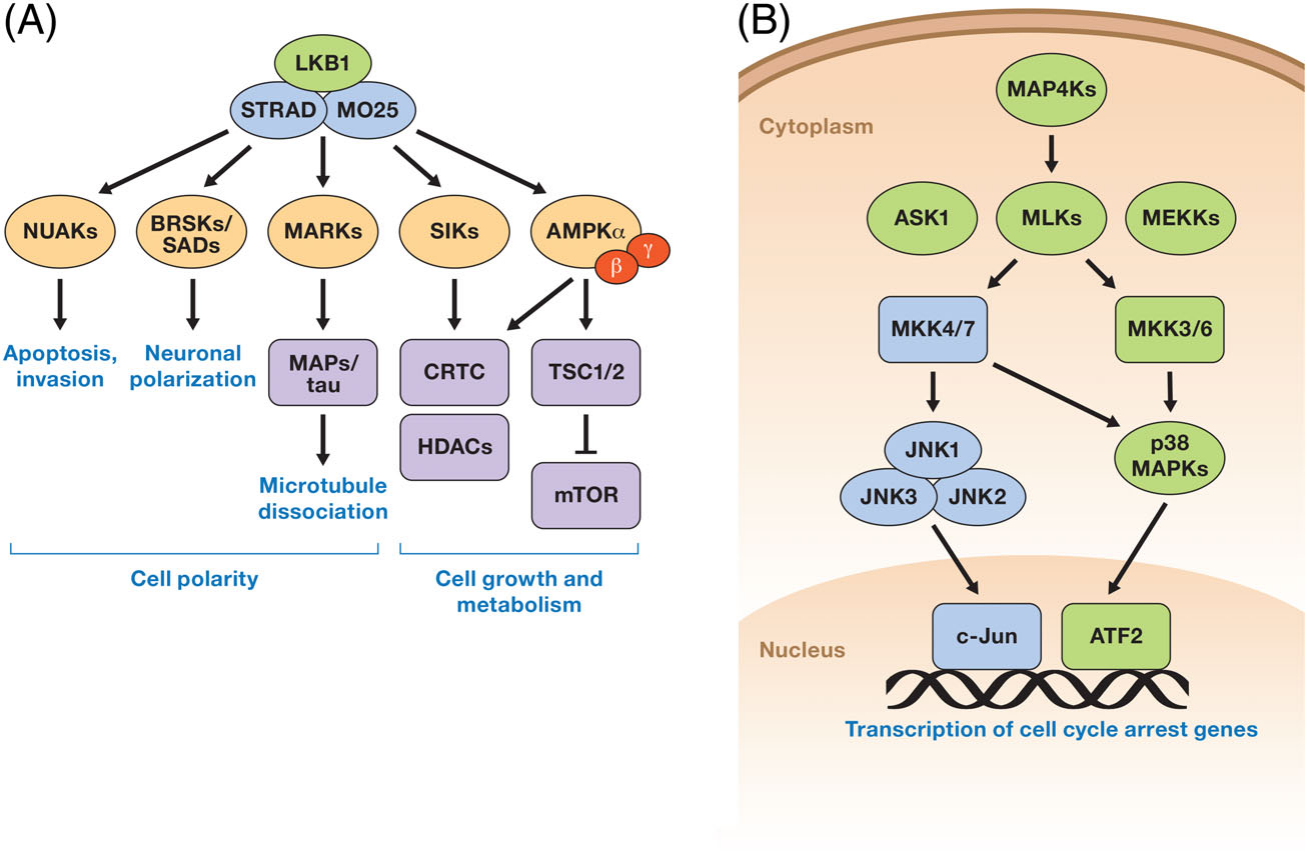
LKB1 and MKK4 tumor suppressors. (A) LKB1, in a complex with STRAD and MO25, directly phosphorylates AMPK and AMPK‐related kinases (NUAKs, BRSKs/SADs, MARKs, SIKs). Activation of these kinases leads to maintenance of cell polarity and negative regulation of cell growth and metabolism. (B) MKK4 phosphorylates and activates JNK1/2/3 and p38 MAPKs. Activation of these kinases leads to activation of transcription factors that regulate the cell cycle and proliferation.

A major mechanism for the tumor‐suppressive function of LKB1 is activation of AMPK and various AMPK‐related kinases (including NUAK1, NUAK2, SIK1, SIK2, and MARK1–4) (Fig. 1A). These kinases all share the conserved T‐Loop phosphorylation site that LKB1 directly phosphorylates to promote a 50‐fold increase in activation and through these kinases LKB1 directly controls numerous cellular processes, including metabolism, growth, and polarity 5, 16, 17. By directly controlling the activation of these kinases, LKB1 inhibits mammalian target of rapamycin (mTOR), a tumor‐promoting kinase, and activates tuberous sclerosis 2 (TSC2) and p53, both of which are tumor suppressors 16, 18, 19, 20, 21. LKB1 activates SIK1 and SIK2, and these kinases phosphorylate transcriptional regulators, including the CREB (cAMP response element‐binding protein)‐regulated transcription coactivator (CRTC) family, and class II histone deacetylases (HDACs) 17, 22 leading to 14–3‐3 binding and cytosolic sequestration of these transcription factors. By promoting the phosphorylation of CRTC and class II HDACs, LKB1 inhibits cellular metabolism. In addition, LKB1 directly activates NUAK1 to regulate the activity of myosin phosphatases, through phosphorylation of myosin phosphatase targeting‐1 (MYPT1). Phosphorylation of MYPT1 promotes the binding of MYPT1 to 14–3‐3 proteins and suppresses the phosphatase activity of PP1β leading to an increase in myosin light chain 2 (MLC2) phosphorylation and loss of cell adhesion, which can be a hallmark of metastatic cancer cells 23.

It is important to point out that although LKB1 is one of the major upstream activators of AMPK, Ca^2+^—and calmodulin‐dependent protein kinase kinase 2 (CAMPKK2) has also been reported as an activator of AMPK via Thr‐172 phosphorylation. AMPK has two isoforms of the alpha subunit, AMPK*α*1 and AMPK*α*2, which have differential and overlapping functions in various cell types. More recent findings have shown that treating LKB1‐null tumor cell lines with Ca^2+^ ionophore A23187 (activator of CAMPKK2) causes a G1 arrest similar to that caused by LKB1 re‐expression 24. Fogarty et al. showed that this phenotype can be prevented by expressing a dominant‐negative AMPK mutant or by double knockout of both AMPK alpha subunits suggesting that AMPK activity is needed for cell‐cycle arrest 24.

In summary, LKB1 was one of the first kinases to have displayed predominantly tumor‐suppressive properties. Its identification as a tumor suppressor contributed to negating the preconceived notion that kinases possess only pro‐tumorigenic activity.

### MKK4: A Dual Specificity Tumor‐Suppressing Kinase

Another tumor‐suppressing kinase is mitogen‐activated protein kinase kinase 4 (MKK4). MKK4 is a dual specificity kinase, which means it has both tyrosine kinase and serine–threonine kinase activity. MKK4 is encoded by the *MAP2K4* gene located on human chromosome 17. Environmental stress, cytokines, and peptide growth factors activate MKK4 25. MKK4 was first identified in screens for MKK family members in *Xenopus laevis* and termed XMEK2 26. Homologs in *Drosophila melanogaster* (DMKK4) and humans were later cloned 27, 28, 29

The role of MKK4 as a tumor suppressor came from an effort to discover homozygous deletion events in human cancer cell lines. This approach was taken on the basis of success in localizing tumor‐suppressor genes by analyzing chromosomes for sites of deletion breakpoints 30. In a pancreatic cancer cell line, *MAP2K4* mapped with the D17S969 marker, which is located in a region of high incidence of LOH in multiple cancers 31. Subsequent positional cloning revealed a homozygous deletion in *MAP2K4*, indicating MKK4 may be a novel tumor suppressor.

MKK4 phosphorylates and activates Jun‐N‐terminal kinases (JNKs) and p38 family of kinases, leading to regulation of various transcription factors including c‐Jun (Fig. 1B). Some of the tumor‐suppressive functions of MKK4 can be attributed to its activation of these kinases 32, 33, which regulate tumor‐suppressing signaling cascades 34, 35. Indeed, LOF mutations and deletions in kinases in the JNK and p38 signaling cascades are also associated with various cancers 31, 36 (Fig. 1B).

Further supporting a tumor‐suppressive role of MKK4, a genomic study mining for mutations in the human kinome in 356 tumor samples identified 11 tumors that contained somatic mutations in the kinase domain of *MAP2K4* 37, 38, 39. Biochemical analysis of the resulting MKK4 mutants showed that these mutations were predominantly LOF 40. Homozygous loss of *MAP2K4* often co‐occurred with *TP53* (encoding p53) and *KRAS* mutations in lung adenocarcinomas, suggesting there are specific genetic backgrounds in which MKK4 functions as a tumor suppressor. Furthermore, studies with mouse *Kras*‐*Tp53*‐mutant lung adenocarcinoma cells and human pancreatic cancer cells showed that increased expression of MKK4 decreased invasive behavior in culture and restoring expression of MKK4 in the *Kras*‐*Tp53*‐mutant mouse cells reduced their metastasis when injected in mice 40.

One mechanism by which MKK4 controls invasive behavior is independent of its activation of JNK and p38 kinases. Inhibitors of these kinases did not affect invasive behavior. Instead, the enhanced invasive behavior of the mouse *Kras*‐*Tp53*‐ mutant lung adenocarcinoma cells deficient in MKK4 depended on the increased abundance of peroxisomal proliferator‐activated receptor γ2 (PPARγ2) 40. Thus, MKK4 has a tumor‐suppressive role in lung adenocarcinoma that involves diminished tumor cell invasion through downregulation of PPARγ2.

Another potential mechanism by which MKK4 can function as a tumor suppressor is through promotion of senescence, which is a commonly recognized mechanism of tumor suppression. Senescent fibroblasts have increased abundance of MKK4 41. In addition, MKK4 overexpression stimulates a senescent phenotype in WI‐38 human fibroblasts, whereas MKK4 depletion suppresses the senescent phenotype 41.

However, there is some controversy regarding the role of MKK4 as a tumor suppressor. Some studies have reported an oncogenic function for the kinase. For example, skin‐specific MKK4‐deficient mice are resistant to carcinogen‐induced tumorigenesis 42. Tumor‐promoting roles of MKK4 have been reported in human breast and pancreatic cancers 42. Adenoviral expression of MKK4 in MKK4‐deficient cell lines stimulated proliferation and invasive behavior, but MKK4 abundance far exceeds the endogenous amounts in these experiments. Lastly, MKK4 knockdown with silencing RNA (siRNA) in a MKK4‐positive breast cancer cell line resulted in decreased anchorage‐independent growth, increased apoptosis in serum‐deprived conditions, and suppressed tumor growth in a mouse xenograft model, indicating that in some genetic contexts MKK4 can promote tumorigenic phenotypes.

This is a common theme for many kinases: They can act both in tumor‐suppressive and tumor‐promoting capacities, depending on the genetic make‐up of the tumor 43. The identification of predominantly LOF mutations and deletions in numerous human cancers indicate that, for MKK4, the kinase predominantly has tumor‐suppressing role in many cancers.

It is important to mention that some of the very first LOF mutations in a protein kinase were discovered in ataxia‐telangiectasia mutated (ATM) kinase. Early studies of ataxia‐telangiectasia (A‐T), a genetic immunodeficiency disease caused by mutations in ATM, suggested a link between A‐T and cancer predisposition 44. Supporting the role of ATM as a tumor suppressor in cancer, a high frequency of loss of heterozygosity at 11q22‐q23 (a locus that includes ATM) is observed in female breast cancer 45 and a high frequency of ATM LOF mutations is found in T‐cell prolymphocytic leukemia (T‐PLL) 46. Interestingly, different mutations can lead to the development of unique diseases based on loss of expression compared to loss of activity and include lack of coordination or neurodegeneration; predisposition to leukemia and lymphoma; immunodeficiency; and hypersensitivity to ionizing irradiation 47.

## USING CANCER GENOMIC DATA TO DEFINE NOVEL TUMOR‐SUPPRESSING KINASES

**Figure 2:**
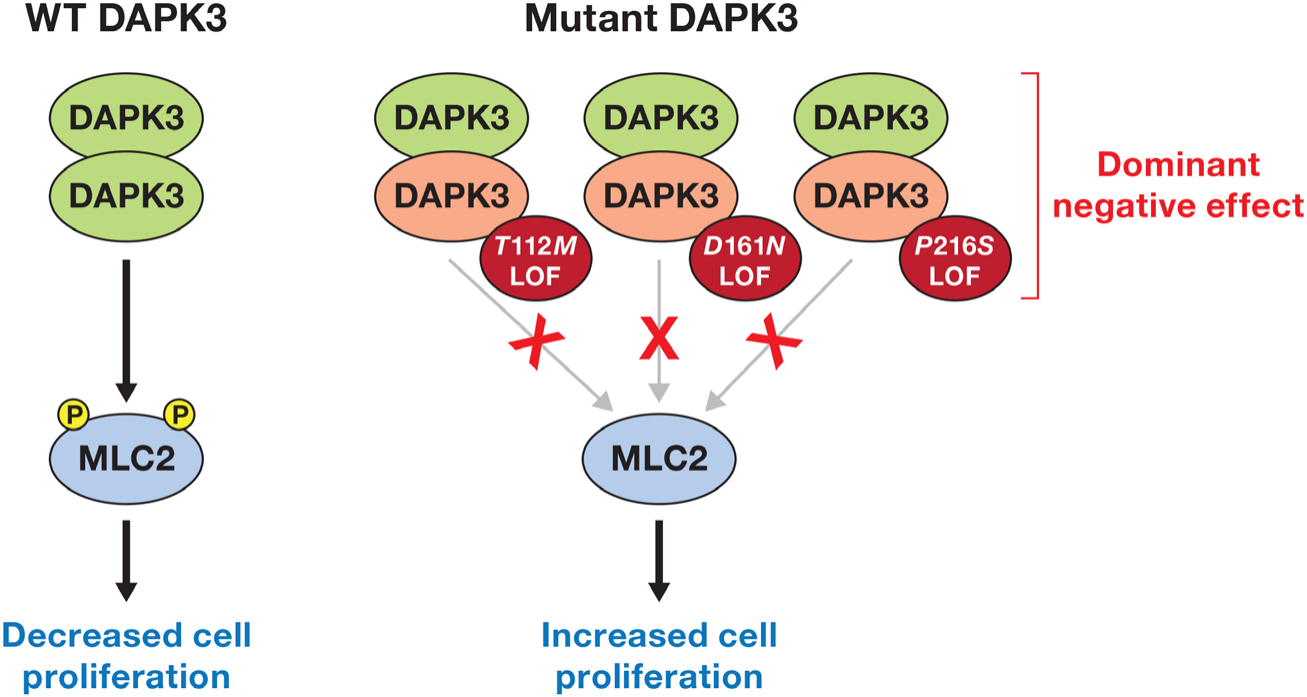
Suppression of tumorigenesis by DAPK3. Active wild type DAPK3 dimers directly phosphorylate MLC2 to suppress migration and proliferation and promote cellular adhesion. DAPK3 mutants act in a dominant negative manner by dimerizing with WT DAPK3 resulting in inactivation of the WT allele and suppression of MLC2 phosphorylation. Unphosphorylated inactive MLC2 in turn leads to sustained cellular adhesion, increased cell survival, and drug resistance.

**Figure 3:**
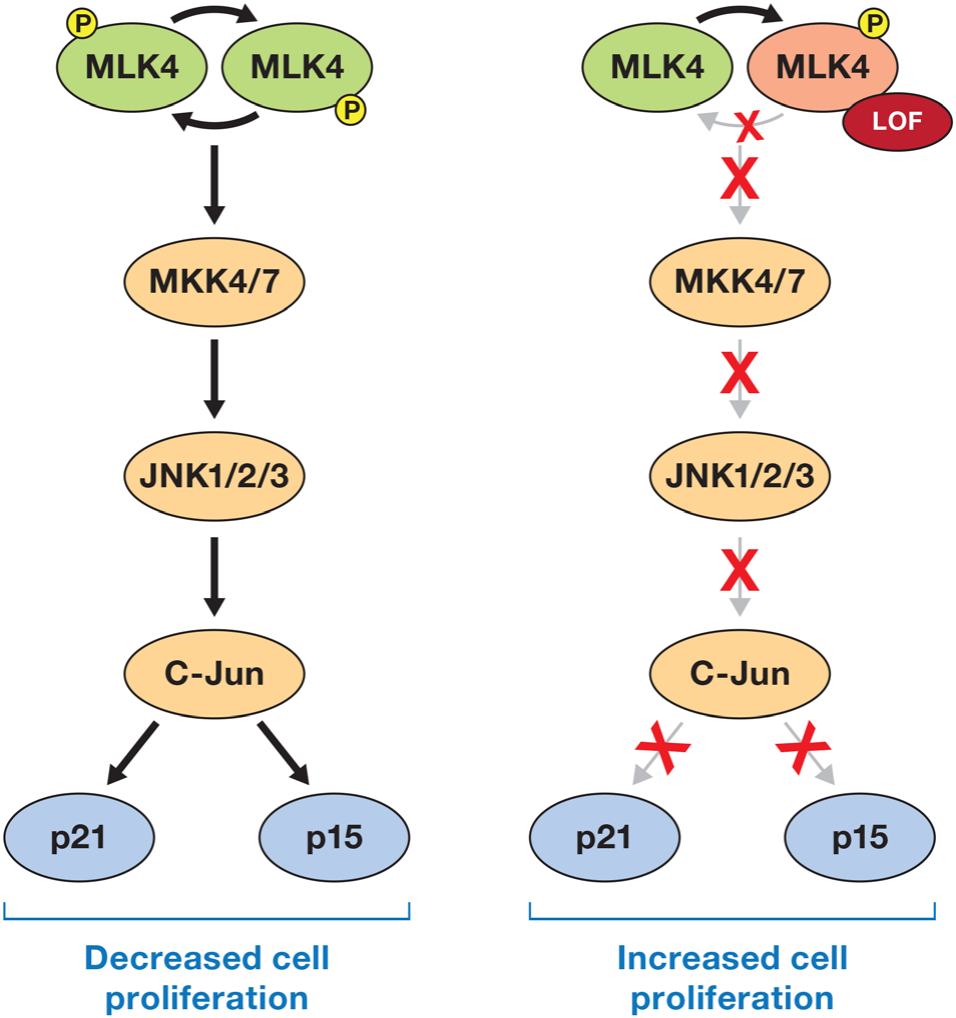
MLK4 activates the JNK pathway leading to increased expression and stabilization of cell cycle inhibitors p21 and p15 to suppress cellular proliferation. LOF mutations in MLK4, similar to DAPK3, act in a dominant negative manner to suppress activation of WT MLK4. This results in inactivation of the JNK signaling cascade and decreased abundance of p21 and p15 and consequently increased cell proliferation.

Tumor suppressors display distinct mutational signatures that include missense, nonsense, and frame‐shift mutations, deletions, and insertions, each of which can inactivate the encoded tumor‐suppressing protein. In general, two copies of a gene are necessary for its normal function, thus, losing one copy leads to haploinsufficiency. Deletion of a second allele, or homozygous deletion, leads to complete loss of expression of a gene product and is commonly observed for tumor suppressors. Alternatively, instead of complete loss of an allele, somatic mutations in a single allele can compromise catalytic activity of an enzyme. If dimerization followed by autophosphorylation is required for activation of a kinase, then loss of catalytic activity of a single allele can suppress the activity of the WT allele, resulting in a dominant negative effect and loss of 75% of overall enzymatic activity for the mutated protein within a cancer cell. Examples of heterozygous LOF mutations resulting in a dominant negative effect include DAPK3 and MLK4 48, 49 (Figs. 2 and 3).

For classic tumor suppressors, the presence of a homozygous LOF mutation will lead to complete loss of function for the tumor suppressing protein. In addition, compound heterozygous mutations that are often observed in tumor suppressors, result in LOF of both alleles through unique mechanisms. An example of biallelic compound heterozygous mutations is evident in ATM kinase where the two mutations occur in two unique locations for each allele (5573G > A and 6154G > A) but lead to LOF of both alleles. In this example, the 5573G > A (Trp1858*) mutation is a truncation mutation resulting in loss of the kinase domain, and the 6154G > A (E2052K) is a missense mutation within the kinase domain which leads to loss of catalytic activity 50.

Activating oncogenic mutations generally occur at specific residues with a high frequency across cancers, examples include the G12 residue in KRAS, the V600 residue in BRAF, and the L858 residue in epidermal growth factor receptor (EGFR). However for tumor suppressors, somatic mutations are generally spread across the entire gene as numerous mutations can lead to LOF or loss of expression of the tumor suppressor. Tumor suppressors that are proteins with enzymatic functions will acquire mutations in conserved residues that are required for catalytic activity. These mutations would compromise rather than cause constitutive activation. Using various bioinformatic tools, such as Mutation Assessor, SIFT, KinView, Kin‐Driver, and Polyphen, researchers can predict if a mutation is pathogenic. Because evolutionary conservation is a key component of bioinformatic prediction algorithms, they are biased toward predicting LOF mutations in highly conserved residues. Kinases have numerous residues across the kinase domain that are highly conserved and critical for catalytic activity, consequently, these algorithms effectively identify “functional or pathogenic” mutations that result in loss of activity 51. Therefore, application of high‐throughput bioinformatic algorithms that predict the functional impact of mutations across large sets of sequencing data will predict “pathogenic” mutations that are enriched for LOF mutations in kinases.

### DAPK3: Bioinformatics Driving the Discovery of Driver Mutations

Using the bioinformatic prediction algorithm CanPredict, death‐associated protein kinase 3 (DAPK3) was identified as a cancer‐associated kinase, where every mutation was predicted to be a pathogenic mutation 48. These mutations included heterozygous missense mutations (T112 M, D161N, and P216S). Biochemical characterization of these mutants indicated that all mutants lacked catalytic activity in *in vitro* kinase assays and when overexpressed in cells. Furthermore, the inactive allele acts in a dominant negative manner to suppress activity of the wild‐type allele, because dimerization and autophosphorylation are required for DAPK3 activity (Fig. 2). In addition, studies in a 3D culture system with mouse cells supported a potential tumor‐suppressing function for DAPK3 52. DAPK3 loss increased the proliferation and death of cells that form glandular structures called acini in a 3D culture model and sensitized the cells to signals that stimulate mTOR activation 52. Additionally, in prostate cancer, an inverse relationship exists between AKT abundance and DAPK3 abundance 53, where Akt inhibition or DAPK3 overexpression in cultured prostate cancer cells reduced proliferation of the cells. Thus, one of the tumor‐promoting activities of AKT is repression of the tumor‐suppressing kinase DAPK3.

Defining DAPK3 as a tumor suppressor with LOF mutations in cancer patients was one of the first attempts to use bioinformatics to identify functional mutations in a kinase from cancer genomics studies. This provided clear evidence of the potential of cancer genomics data to define novel tumor suppressing enzymes 48.

### MLK4: MLKing Bioinformatics Pipelines for New Tumor Suppressors

Another example of bioinformatics guiding the identification of novel cancer‐associated kinases enriched in predicted pathogenic mutations comes from the study of mixed‐lineage kinase 4 (MLK4 encoded by *MAP3K21*). This family of kinases was first identified in a study of mRNA expressed in human epithelial tumor cells 54. The MLK family members are characterized by the presence of signature sequences for both serine–threonine and tyrosine kinases within their catalytic domain and serve as MAP3Ks to phosphorylate and activate MKK4/7 and MKK3/6 to activate the JNK and p38 pathway, respectively 55.

Genomic profiling of colorectal cancers (CRCs) identified MLK4 as the second most frequently mutated protein kinase in the CRC subtype called microsatellite stable (MSS) CRC, which has a relatively stable unchanging genome compared to other types of CRC 56, 57. The presence of mutated alleles of *MLK4* in CRC increases the transformation and tumorigenic capacity of RAS‐mutated cell lines 58. Evaluation of the functional impact of the mutations in MLK4 indicated they may have increased kinase activity compared to WT MLK4, however, the WT MLK4 displayed similar enzymatic activity as a kinase dead mutant in these studies, indicating the WT MLK4 they used in their assays may have had an additional mutation that abolished catalytic activity. Consistent with this possibility, it was later demonstrated that a majority of mutations in MLK4 abolish catalytic activity and this was repeated by three sets of independent researchers located at different research institutions, unequivocally demonstrating that mutations in MLK4 abolish catalytic activity 49. Furthermore, MLK4 LOF mutants can act in a dominant negative manner to suppress the activation of the WT allele highlighting why most LOF mutations in MLK4 are heterozygous as a single LOF mutation in an MLK4 allele can abolish a majority of signaling downstream of this kinase 49 (Fig. 3). Reconstitution of CRC cells harboring a LOF mutation in MLK4 with WT MLK4 at levels that are able to overcome the dominant negative effect suppressed CRC cell proliferation by directly activating MKK7 leading to activation of JNK1/2 49. JNK1/2 activation led to an increase in cJUN protein levels and ultimately led to an increase in expression of the cell cycle inhibitors p21 and p15, providing the molecular mechanism by which MLK4 suppresses CRC cell proliferation and why LOF mutations in MLK4 will be beneficial to CRC cell proliferation 49. The possibility still exists that mutations in MLK4 could be neomorphic and suppress signaling in canonical MLK4 pathways (LOF) but may display an unknown GOF towards other pathways, either as a scaffold protein or possibly the mutants may display an altered substrate specificity.

## CHALLENGING THE TUMOR‐PROMOTING DOGMA THROUGH SYSTEMATIC EVALUATION

Analysis of the role of EPH receptors in cancer illustrates the complexity in inferring tumor‐suppressing or tumor‐promoting function from transcript expression data. Historical data can also lead to inaccurate interpretations as exemplified by the classification of members of the protein kinase C (PKC) family as tumor promoters. These examples illustrate how dogma can be difficult to overturn and the need for systematic evaluation of cancer mutants identified in unbiased cancer genomic sequencing studies to define the role of specific kinases in cancer.

### Ephacing an Oncogenic Role for Eph Receptors in Cancer

The erythropoietin‐producing human hepatocellular carcinoma (Eph) receptors were first identified in 1987 during a search for tyrosine kinases involved in cancer 59. A human genomic library was searched for gene sequences homologous to the tyrosine kinase domain of the viral oncogene *v‐fps*, which was overexpressed in an erythropoietin‐producing human hepatocellular carcinoma cell line (ETL‐1). Eph receptors constitute the largest family of receptor tyrosine kinases. They interact with a group of eight ligands called Ephrins (Eph receptor‐interacting proteins), which can be divided into two types: the B‐type which are transmembrane proteins with extracellular receptor‐binding domain and short cytoplasmic tails required for reverse signaling, and the A‐type which are small proteins containing only a receptor‐binding domain linked to the membrane via glycosylphosphatidylinositol (GPI) anchor, but can also transmit reverse signals when bound to Eph receptors 60. Thus, both Eph and Ephrins engage signaling cascades in their respective cells: The signal mediated by Eph receptors is called “forward signaling” and the signal mediated by Ephrins is called “reverse signaling.” In many cancer cell lines, Eph receptors are highly expressed 61; however, the receptors are poorly activated based on the low levels of phosphorylation that are detected 61. This low level of phosphorylation hinted that ephrin‐dependent Eph forward signaling might be tumor suppressive. Consistent with a tumor‐suppressive function for these receptors, some of these RTKs inhibit oncogenic signaling pathways, including the HRAS‐ERK, PI3K‐AKT, and ABL‐CRKL pathways 61.

Experiments with ephrin‐A1‐Fc fusion protein, a soluble chimeric protein that activates EPHA2, showed that EPHA2 has tumor‐suppressing activity. Activation of EPHA2 receptors with ephrin‐A1‐Fc fusion protein decreases migration, invasion, survival, and proliferation of various types of cancer cells in vitro and in vivo 62, 63, 64, 65, 66. Stimulation of EPHA2 with the fusion protein reduces ERK activation 62 and attenuates phosphorylation of ERK in response to other growth factors, such as epidermal growth factor (EGF). Inhibition of the transformation of NIH3T3 cells by a viral oncogene, *v‐ERBB2*, by EphA stimulation also supports a tumor‐suppressing role 61. EPHA2‐deficient mice display increased susceptibility to chemical carcinogen‐induced skin cancer, which is accompanied by increased tumor cell proliferation and phosphorylation of ERK 67. These data indicate that ephrin‐A‐induced EPHA forward signaling inhibits tumor malignancy. In summary, ephrin‐induced EPHA receptor forward signaling represents a tumor‐suppressing activity. However, upon tumor initiation, Eph receptor abundance is upregulated by oncogenic signaling pathways, such as the RAS/MAPK pathway in breast cancer, or the Wnt‐β‐catenin pathway in colon cancer. In contrast, their ephrin ligands are often down‐regulated or through a loss of cell contact the ephrins will not bind to the receptors, thus EPHA forward signaling is impaired. Loss of EPHA signaling enables enhanced activation of oncogenic pathways, HRAS‐ERK, PI3K‐AKT, and ABL‐CRKL.

Not all Eph receptors may exhibit a net tumor‐suppressive effect, however. For instance, EphB2 enhances proliferation and suppresses invasiveness in mouse intestinal progenitor cells and Apc^min/+^ adenomas 68. The increase in proliferation involves an ABL1‐mediated increase in cyclin D1, which stimulates progression through the cell cycle. The net effect is tumor promoting rather than tumor suppressing.

High‐throughput screens of tumor samples and cell lines have identified numerous somatic mutations in nearly all Eph receptors 38, 69, 70. Moreover, *EPHA3* is one of the most frequently mutated members of the Eph family, with numerous missense mutations in lung cancer 71. A systematic characterization of 28 reported mutations in EPHA3 showed that many were detrimental to kinase activity, autophosphorylation, cellular trafficking, or ephrin binding 71. Thus, these would impair activity, indicating that loss of EPHA3 activity will lead to cancer development and provide compelling evidence for a tumor‐suppressive role of EPHA3 in cancer.

Other members of the Eph family have displayed tumor‐suppressive functions in various cancers, including EPHB4 in colorectal and prostate cancers 64, EPHB6 in androgen‐deprived prostate cancer 72, and EPHA5 in colorectal carcinoma 73. Thus, many members of the Eph family have tumor‐suppressive roles in human cancers.

### PKCs: Reversing the Paradigm

The PKC family of kinases includes the conventional PKC isoforms (α, β, and γ) with functional C1 and C2 domains, which bind diacylglycerol (DAG) and calcium, respectively, to promote membrane translocation and activation; the novel family members (δ, ε, η, and θ) with a functional C1 domain and with a nonfunctional C2 domain; and the atypical family members (ζ and ι), which lack functional C1 and C2 domains and are primarily regulated through protein–protein interactions. Because PKCs serve as the primary receptor for phorbol esters, which are tumor promoters that enhance carcinogenesis in skin cancer models 74, PKCs were initially classified as tumor promoters. Similar to diacylglycerol, phorbol esters bind the C1 domain of conventional and novel PKCs. However, phorbol esters are not easily metabolized, leading to hyperactivation of these PKC isoforms. The hyperactivation ultimately leads to PKC dephosphorylation and degradation. Consequently, although initial activation of PKCs by phorbol esters was a reasonable explanation for the tumor‐promoting properties of phorbol esters, long‐term degradation and loss of signaling by the conventional and novel PKCs may be the main mechanism driving tumorigenesis.

To conclusively determine if PKCs are tumor suppressors or oncogenes, a systematic approach was taken to evaluate the functional impact of mutations in PKCs and determine if mutations in PKCs alter their respective enzymatic activity 75. Biochemical analysis of 46 mutations in PKC isozymes that are present in many different tumors revealed that most mutations are LOF. Indeed, none are GOF. Excluding insertions, deletions, or truncating mutations, two‐thirds of somatic mutations in PKCs are inactivating. Various mechanisms of inactivation were described including disrupting the catalytic site, preventing second messenger binding, or impairing phosphorylation. Bioinformatic analysis using mutations in conserved residues required for catalytic activity as the criteria for LOF identified additional PKC mutations that abolish catalytic activity 76. In addition, various truncating mutations in PKCs have been described in cancers. Although not all have been assessed for biochemical activity, many are predicted to result in LOF. Using KinView, a visual comparative sequence analysis tool, additional LOF mutations in PKCs were identified 76. Not all mutations simply abolish catalytic activity. Some are neomorphic, generating new functions for the protein. These types of mutations can convert a tumor‐suppressing protein into a tumor‐promoting one. For instance, in lung cancer, a PKCγ mutation changes the substrate specificity of the enzyme 77.

Strikingly a single LOF mutation in a PKC isozyme not only affects the mutated enzyme, but can also suppress the activity of other PKC isozymes. In an eloquent set of experiments with the DLD1 colon cancer cell line harboring a LOF mutation in PKCβ (A509T, located in the conserved APE motif) demonstrated the broad impact of a single LOF PKC mutation 75, 78. Correction of the mutant allele by genome editing suppressed anchorage‐independent growth and tumor growth in vivo. Additionally, not only was PKCβ activity restored, the activity of other PKC isozymes increased when this single PKC LOF mutation was corrected, suggesting that the PKCβ A509T mutant exerted a dominant‐negative activity toward other PKC isozymes. In support of a dominant‐negative activity of mutant PKCs, a LOF mutation in a single PKC‐encoding gene prevents processing of other PKCs by reducing the availability of PKC regulators, such as PDK‐1 79, 80. Therefore, a single LOF mutation in a PKC isoform can suppress signaling by multiple PKC isoforms and act in a hyperdominant‐negative manner towards other conventional and novel PKC isozymes.

## DETERMINING THE TUMOR‐SUPPRESSING KINOME

**Figure 4:**
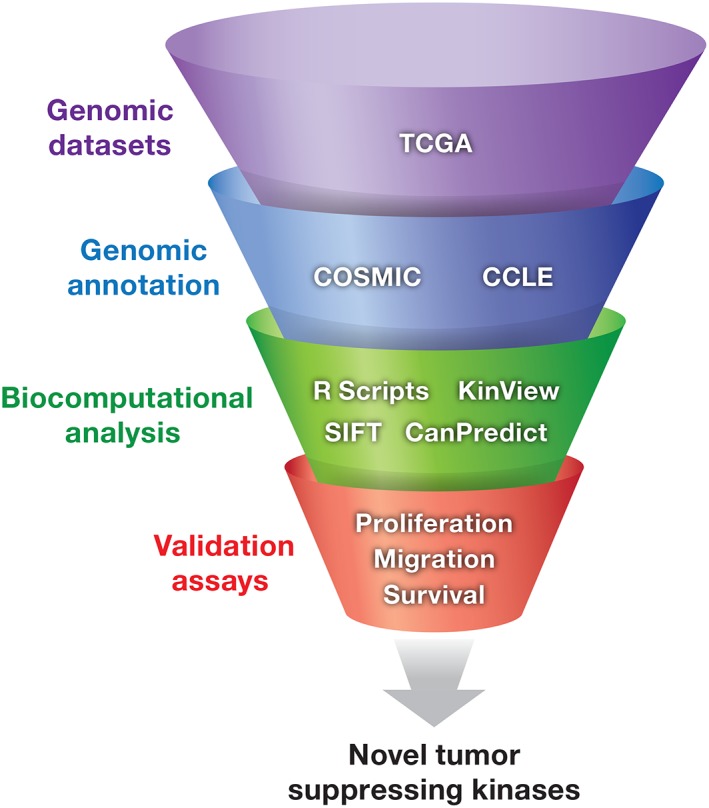
The Tumor suppressing kinome. Filtering of Cancer genomic datasets with high‐throughput mutation assessors that are biased toward predicting functional mutations in highly conserved residues or R scripts designed to identify mutations in critical residues required for catalytic activity greatly aid in identifying novel tumor suppressing kinases. Validation is then required through biochemical and functional assays.

Determining genomic aberrations that drive tumor biology and implementing this knowledge to guide precision medicine‐oriented clinical trials is one of the main focuses in cancer research. The distribution of disease‐driving mutations within kinases is not random, and machine‐learning approaches can be used to identify mutations with functional consequences 81, 82, 83. In a global approach to define the tumor‐suppressing kinome, we mined the Cancer Genome Atlas (TCGA) and the Cancer Cell Line Encyclopedia (CCLE) datasets to generate a list of candidate tumor‐suppressing kinases (Fig. 4). We used the frequency of truncating mutations, which would abolish catalytic activity, as the criteria for inclusion as a putative tumor‐suppressing kinase 84. By aligning the top 30 candidates from this screen, we established a conservation score for every amino acid for these top 30 tumor suppressing kinases 84. We evaluated mutational frequency at highly conserved residues to identify amino acids that had not previously been considered critical for the catalytic function of a kinase and were mutated at a high frequency 84. The top 12 identified mutational hotspots were part of the highly conserved motifs (APE, HRD, and DFG) required for protein kinase catalytic activity, validating that this approach identifies residues important for catalytic activity. We identified two new hot‐spot residues, at the sixth position before the APE motif and the sixth position before the HRD motif, that abolished kinase activity. PKCθ was among the kinases with mutations in these residues.

Expanding the analysis to kinases enriched in mutations in the top 15 hotspot residues and ranking the mutant kinases based on the frequency of their occurrence in cancer not only identified known tumor‐suppressing kinases but also identified previously unknown candidate tumor‐suppressing kinases 84. STK11, EPHB1, and CHEK2, which are known tumor‐suppressing kinases, were some of the kinases meeting the expanded criteria 85, 86, 87, 88. From the novel candidates, MAP2K7 exhibited a high occurrence of mutations in a specific type of cancer, gastric adenocarcinoma 84. Biochemical studies of the cancer‐associated hotspot mutations in MAP2K7 indicated that most are LOF 84. As a kinase in the JNK signaling pathway, LOF of MAP2K7 supports the tumor‐suppressing properties and inactivation of the JNK signaling cascade in gastric cancer 84. This type of analysis provides a foundation for the development of other bioinformatics screens to investigate properties and disease associations for enzymes outside of the human kinome.

## KINASES IN CANCER: THE FUTURE

With the onset of fully annotated human genome, present‐day biology has transitioned into an era of ever expanding data from high‐yield cancer genomic studies. The field of bioinformatics has rapidly evolved to efficiently mine genomic data sets with web‐based tools, such as KinView, Mutation Assessor, SIFT, and Polyphen. Specifically, KinView provides a platform for comparative analysis and visualization of a protein kinase to determine posttranslational modifications 76. This tool enabled the identification of variable phosphorylation patterns in the kinase domains of serine/threonine kinases and tyrosine kinases. These tools also support molecular modeling to aid in the prediction of the functional effect of mutations.

Functional studies are essential in validating potential LOF mutations in novel tumor suppressing kinases and many intriguing targets have recently been identified. For example, vertebrate class III myosin A (MYO3A) was identified as one of the top kinases harboring frequent truncation and potential LOF mutations in numerous cancer types 84. Little is known regarding MYO3A as it is an understudied kinase, but the genetic data leaves little doubt the kinase will play an important role in suppressing tumorigenic phenotypes 84. Numerous additional understudied and novel kinases were also revealed by Hudson et al. through the high‐throughput tumor suppressing screens, indicating we are just scratching the surface in fully understanding the numerous mechanisms utilized by cancer cells to promote tumorigenesis 84 (Table 1).

**Table 1:** List of validated tumor suppressor kinases^a^

LKB1	GSK3B	STK10/11
ATR	MAP3K4	SYK
BMPR2	MAP3K21	TNK1
BTK	NME1	LATS1
CHEK1	NTRK3	NUAK1
CHUK	ROR2	MAP4K1
MAP3K8	DDR2	MAP2K7
CSNK1A1	PRKAA1/2	CHEK2
DAPK1	PRKAR1A	DAPK2
DAPK3	PRKCB/D/E	LATS2
DOK1	MAPK9/10	RPS6KA6
FRK	RPS6KA2	HIPK2
PLK5	MAP2K4	CAMK2N1
SIK1	BRSK1	WNK2

a Data extracted from Tumor Suppressor Gene Database (https://bioinfo.uth.edu/TSGene/) and the literature.

Surprisingly, the high‐throughput tumor‐suppressing screen conducted by Hudson et al. revealed the presence of LOF mutations in known oncogenic kinases 84. There is precedence for LOF mutations in oncogenic kinases and for these LOF mutations to promote tumorigenesis. For example, LOF mutations in the oncogenic kinase BRAF can paradoxically promote the activation of the MEK–ERK cascade in the presence of genetically activated upstream regulators of BRAF, such as RAS or EGFR 86. In this genetic environment, the inactivated BRAF allele acts as a scaffold to promote the activation of CRAF, thereby hyperactivating the MEK–ERK pathway 86. LOF mutations were identified in EGFR and SRC, two well‐characterized oncogenic kinases 84. The effect of such mutations on the functions of these kinases in cancer remains unknown. It is possible that the inactive EGFR may promote the activity of the wild‐type receptor, in a manner similar to inactive ERBB3 promoting activation of EGFR 89, 90. This would be an important discovery, because it suggests that patients with LOF mutations in EGFR may benefit from treatment with already clinically approved EGFR inhibitors.

As we continue to expand our “omics” technologies, combining multiple datasets with high‐throughput mutational screening approaches will provide a platform for discovering a vast array of important tumor promoters and tumor suppressors present in the “tail” (genes with a lower frequency of mutations) of cancer genomics studies (Fig. 4). This information will ultimately lead to new mechanisms of tumorigenesis and the development of novel cancer therapies.

## CONFLICT OF INTEREST

The authors declare no potential conflicts of interest.
